# Barriers and Facilitators to Delivering Cancer Care in US Prisons

**DOI:** 10.1001/jamanetworkopen.2025.37646

**Published:** 2025-10-15

**Authors:** Christopher R. Manz, Brett Nava-Coulter, Emma Voligny, Daniel A. Gundersen, Alexi A. Wright

**Affiliations:** 1 Department of Medical Oncology, Dana-Farber Cancer Institute, Boston, Massachusetts; 2 Harvard Medical School, Boston, Massachusetts; 3 Rutgers Institute for Nicotine and Tobacco Studies, New Brunswick, New Jersey; 4 Department of General Internal Medicine, Rutgers Robert Wood Johnson Medical School, New Brunswick, New Jersey

## Abstract

**Importance:**

Cancer is the leading cause of death in US prisons. Incarcerated patients with cancer have higher mortality than the general population, but little is known about barriers and facilitators to cancer care in prison.

**Objective:**

To describe barriers and facilitators to cancer care in US prisons from screening and diagnosis to end-of-life care.

**Design, Setting, and Participants:**

Semistructured qualitative interviews were conducted among clinicians involved in delivering cancer care in US prisons, including prison medical directors, primary care practitioners (PCPs), and oncologists in 16 US state and federal prison systems between September 2023 and April 2024, and a member-checking focus group was conducted in February 2025. Data were analyzed from September 2023 to February 2025.

**Main Outcomes and Measures:**

The main outcomes were identification of key themes regarding barriers and facilitators to delivering cancer care to incarcerated patients across US prison systems. Interview transcripts were analyzed using mixed inductive and deductive thematic content analysis to identify emerging themes.

**Results:**

A total of 32 participants from 16 prison systems were interviewed, including 9 medical directors, 6 PCPs, and 15 oncologists, with a median (range) age of 49 (33 to ≥70) years. Participants predominantly self-identified as female (19 participants [59%]); 6 (18.8%) as Asian or Pacific Islander; 5 (15.6%) as Black, African, or African American; and 21 (65.6%) as White. Participants described 7 themes as barriers: (1) patients’ acceptance of care; (2) population health and determinants of health; (3) care coordination between prisons and oncology practices; (4) communication between patients, oncologists, and prison clinicians; (5) symptom management; (6) transportation; and (7) humanity in care; as well as 3 cross-cutting themes of understaffing, prioritization of security over health, and nontransparent care within prisons. Participants described 2 themes regarding incarceration as a facilitator: increased access to health care for individuals with poor access in the community and help overcoming social determinant barriers. The 22-participant focus group affirmed these themes, added additional context, and identified no new themes.

**Conclusions and Relevance:**

In this qualitative study of clinicians involved in cancer care delivery for individuals incarcerated in US prisons, participants identified numerous barriers to cancer care delivery in prisons. These barriers represent opportunities for targeted interventions to improve cancer outcomes.

## Key Points

**Question:**

What are the barriers and facilitators to cancer care in US prisons?

**Findings:**

In this qualitative study, 32 prison medical directors and clinicians reported barriers related to patients’ acceptance of care, population health determinants, care coordination, communication, symptom management, transportation, staffing, prioritization of security over health and humanity, and nontransparent care in prison. They also reported that incarceration facilitates access to health care and overcoming social determinants of health for this population. Findings were affirmed by a focus group.

**Meaning:**

These findings suggest there are numerous barriers to cancer care in prison that may represent opportunities to improve care for this vulnerable population.

## Teaser

This qualitative study evaluates barriers and facilitators to cancer care in US prisons from screening and diagnosis to end-of-life care.

## Introduction

Cancer is the leading cause of death in US prisons.^1,2^ Incarceration at the time of diagnosis is associated with worse cancer mortality.^3^ While there are limited high-quality data on incarceration and cancer outcomes, a rigorous population-level study in Connecticut found that individuals who are incarcerated when diagnosed with cancer have a 92% higher risk of death than those without an incarceration history.^4^ Incarceration compounds preexisting health inequities because it disproportionately affects disadvantaged populations (eg, those with Black race, low income, and unstable housing) who are burdened by worse cancer outcomes.^5,6^ Thus, efforts to improve cancer diagnosis and treatment for incarcerated individuals are essential to reduce cancer disparities.

To our knowledge, researchers have not systematically evaluated barriers and facilitators to cancer care diagnosis and treatment across US prisons. One such study of English and Welsh prisons documented 3 major barriers: (1) communication restrictions between patients and clinicians and between clinicians practicing inside and outside of prisons; (2) limited access to information, supportive care, and emotional support; and (3) security and logistical challenges that disrupt care.^7,8^ Yet a companion registry-based study found that incarcerated patients had a 16% higher risk of death compared with nonincarcerated patients, which is much lower than disparities observed in Connecticut.^4,9^ Barriers to cancer care may be more pronounced in US prisons due to higher incarceration rates and a more fragmented health care system.^2,10^

Previously, we described how cancer care is provided in US prisons, from screening and diagnosis to end-of-life care.^11^ We found that the care that incarcerated individuals receive in prisons differed significantly from cancer care outside of prisons in ways that may contribute to observed survival disparities. In this study, we aimed to identify barriers and facilitators to cancer care delivery in US prisons by interviewing prison medical directors, primary care practitioners (PCPs), and oncology clinicians with direct experience providing cancer care to incarcerated individuals.

## Methods

### Study Overview

We conducted interviews and a focus group with clinicians who provide cancer care for individuals incarcerated in US prisons to understand how it is provided, perceived barriers and facilitators, and ways to improve cancer care in this setting. This manuscript reports findings related to barriers and facilitators of care. The study was approved by the Dana-Farber/Harvard Cancer Center institutional review board and meets Standards for Reporting Qualitative Research (SRQR) reporting guidelines.^12^

### Study Population and Recruitment

The study included (1) prison medical directors, (2) prison PCPs, (3) gynecologists, (4) medical oncologists, (5) radiation oncologists,( 6) gynecologic oncologists, and (7) palliative care clinicians involved in cancer care for individuals incarcerated in US prisons. Participants were recruited via 3 methods (additional details in the eMethods in Supplement 1): (1) we emailed invitations to participants of a Fall 2023 correctional health conference; (2) for 5 selected states that varied by important characteristics (eg, correctional health model), we used purposive sampling to recruit participants via email from each of the 7 clinical roles; and (3) we used snowball sampling of eligible individuals recruited via email who were identified by the research team, colleagues, or study participants as likely to have unique insights into the study questions. We recruited and interviewed participants between August 2023 and April 2024 and offered $200 for participation. Recruitment was stopped when thematic saturation was reached, an additional 5 participants were interviewed, and we completed purposive sampling for the selected states, as described previously.

### Interviews

We developed a semistructured interview guide based on our clinical and research experience in cancer care delivery and correctional health. The guide asked participants to self-report demographics (including gender and race; race was assessed as Black individuals are disproportionately incarcerated in US prisons and clinician perspectives could vary based on race) and covered barriers and facilitators to cancer care in prison compared with the community (eAppendix 1 in Supplement 1; the guide also included other topics reported elsewhere). After obtaining informed consent, C.M. and B.N.C. conducted interviews in person (for 8 American College of Correctional Physicians participants) or via phone or videoconference (24 participants). Interviews were audio recorded and lasted 30 to 60 minutes.

### Focus Group

To member-check the credibility of study findings, an email invitation to participate in a focus group was sent to individuals who participate in a monthly correctional health video conference for prison medical directors and clinicians.^15^ After completion of analysis and initial manuscript preparation, the focus group was held in February 2025. Over 60 minutes, 1 investigator (C.M.) presented the results of the study in 3 sections (logistics, barriers and facilitators, and strategies to improve care) with discussion interspersed between each section (eAppendix 2 in Supplement 1). Investigators (C.M. and A.W.) took notes during the focus group then summarized focus group comments to identify new themes and insights.

### Data Analysis

Audio recordings were transcribed and uploaded to NVivo version 14 (QSR International). We developed a codebook using deductive codes based on the interview guide to describe barriers and facilitators to cancer care delivery. Themes were identified through deductive and inductive thematic content analysis.^13^ B.N.C. coded all interviews while C.M. read all transcripts and coded data. The study team met biweekly, reviewed coded data, resolved differences through discussion, and iteratively revised the codebook with inductive codes from completed interviews. At the completion of coding, B.N.C. summarized findings for each theme with supporting quotes, C.M. wrote the initial draft supported by the summaries and coded data and discussion in team meetings, and the team collectively edited and refined the manuscript. Investigators identified the level of influence (ie, individual, interpersonal, institutional, and policy) associated with each barrier and facilitator, guided by conceptual frameworks for health disparities.^14^ Investigators had no prison incarceration history, and other researcher characteristics were unlikely to influence analysis (eMethods in Supplement 1).

## Results

We enrolled 32 clinicians from 16 state and federal prison systems (Table 1). Participants included 9 prison medical directors (28%),7 radiation oncologists (22%), 6 medical oncologists (19%), and 6 PCPs (19%) with a median (range) age of 49 (33 to ≥70) years. Participants predominantly self-identified as female (19 participants [59%]); 6 (18.8%) as Asian or Pacific Islander; 5 (15.6%) as Black, African, or African American; and 21 (65.6%) as White. Participants described 7 themes related to barriers, 3 cross-cutting barrier themes, and 2 themes related to facilitators (Figure, with supporting quotes in Table 2; eTable in Supplement 1).

**Table 1.** Participant Characteristics

Demographic	Participants, No. (%) (N = 32)
Age, median (range), y	49 (33 to ≥70)
Sex	
Female	20 (62.5)
Male	12 (37.5)
Race	
Asian or Pacific Islander	6 (18.8)
Black, African, or African American	5 (15.6)
White	21 (65.6)
Clinical role	
Prison medical director	9 (28.1)
Prison primary care	6 (18.8)
Radiation oncologist	7 (21.9)
Medical oncologist	6 (18.8)
Gynecologic oncologist	2 (6.3)
Palliative care clinician	1 (3.1)
Gynecologist	1 (3.1)
Prison systems, No.	16
Participants per system	
Texas	5 (14.7)
Indiana	4 (11.7)
Massachusetts	4 (11.7)
North Carolina	3 (8.8)
Rhode Island	3 (8.8)
California	2 (5.8)
Tennessee	2 (5.8)
Federal Bureau of Prisons	1 (2.9)
Florida	1 (2.9)
Idaho	1 (2.9)
Kansas	1 (2.9)
New Jersey	1 (2.9)
Oregon	1 (2.9)
South Dakota	1 (2.9)
Wisconsin	1 (2.9)
Wyoming	1 (2.9)

**Figure.**
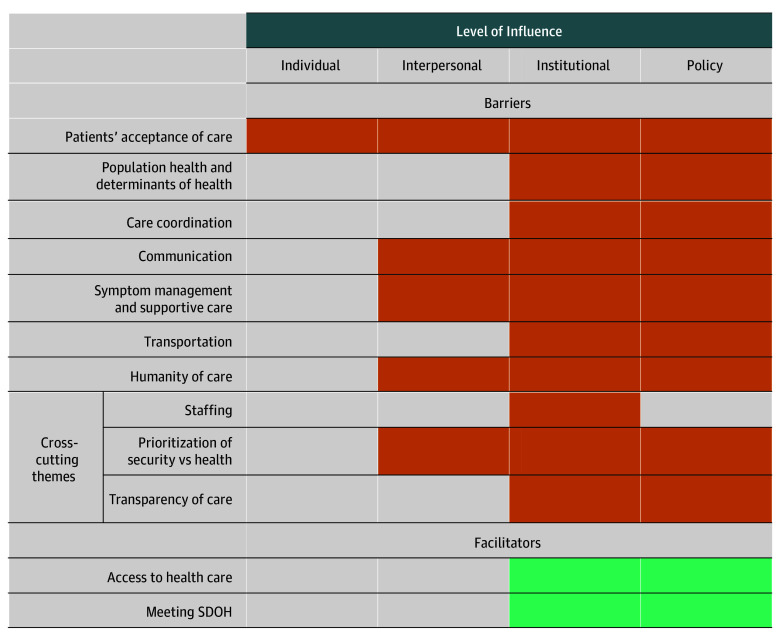
Barriers and Facilitators to Cancer Care by Level of Influence Themes related to barriers and facilitators to care are listed on the left, with colored rectangles indicating the level of influence at which theme acts as a barrier (orange) or facilitator (green). Levels of influence are based on a conceptual framework for health disparities.^14^ SDOH indicates social determinants of health.

**Table 2.** Themes With Supporting Quotes

Barriers	Supporting quotation (participant)
Theme	
Patients’ acceptance of care	“I believe that patients who maybe...would have actually accepted care [if they weren’t in prison] but sometimes they just refuse it. And you can understand it…if they’re in the prison general population…the conditions can be maybe a little bit harsh, and so they don’t want to be weak or perceived as weak or what have you. And so when you say the chemo will probably cause you fatigue and cause you weakness…they are not too open to that.” (Medical oncologist A)
Population health and determinants of health	“In our state, there is delays in diagnosis in part likely because the incarcerated population is also a marginalized population that might have limited access to health care more broadly.” (Medical director C)
Care coordination	“So even though we may heavily suspect cancer, there’s…4 weeks for a colonoscopy, and then another 4 weeks for a CT scan, or another 4 weeks for an interventional radiology biopsy…And this is all kind of like best-case scenario…And realistically, even though we have a high suspicion from beginning to getting seen by an oncologist, we’re looking at 2, 3 months, easy.” (Primary care physician A)
Communication	“One of the biggest issues that I find that’s a huge disparity is how the care is delivered and how stark it is in comparison to the way we practice with patients that are not incarcerated. So, for example, during a conversation about treatment or diagnosis, they’re not allowed to have a family member present. So they’re by themselves, taking in all of this information. If it were anyone else in the community, they can have someone there, take notes, record, ask you questions, recall things. So there’s a complete absence of caregivers when they’re going through this very intense, comprehensive, and overwhelming diagnosis.” (Radiation oncologist F)
Symptom management and supportive care	“Someone that’s being treated from the prison system, even though they’re coming in to the community for care, we can’t give them anything. We can’t even give them a piece of paper, telling them what to use or anything. We have to write it down on paperwork that will be given to the nurse, and it may or may not get delivered to this nurse and by the prison guard. And so these patients can go through treatment in a very agonizing, torturous way.” (Radiation oncologist F)
Transportation	“Right now we’re dealing with custody [that] does not have staffing to take them to appointments. So we were told last month…any new patients can’t get testing. We only have 4 trips out, 4 people a day. We have 3 cancer patients getting radiation almost daily.” (Medical director J)
Humanity in care	“And then finally, any time you’re having a one-on-one with your oncologist, you’re still shackled. You’re still with correctional officers, and so there’s some questions about privacy and confidentiality. Just the image of being in a jumpsuit with shackles affects the provider team with implicit biases that they may or may not be aware of.” (Medical director C)
Staffing	“There are often understaffing of health care systems…at times when we had fewer providers, the priorities were for urgent care visits or other kind of pressing needs before hitting all the health maintenance items. There are other times when we’re well-staffed and it’s very clear that we’re more able to hit the health maintenance reviews.” (Medical director C)
Prioritization	“I do think the prison system is this terrible system, in the sense that the prison was designed to incarcerate people, right? It was not ever designed as a health care delivery mechanism, and it’s not funded as a health care delivery mechanism…And so in some ways, it really does feel like [prisons] are set up to fail [at providing health care].” (Obstetrician gynecologist A)
Transparency of cancer care in prison	“There’s definitely kind of a black box [around administration of symptom medications] that we don’t honestly know, even though we write [it] down what medications are actually given…We kind of make recommendations, but it’s not really like we have a MAR [medication administration record] or medical medication record that we can look at and see if that actually was provided at some time.” (Medical oncologist C)
Facilitators	
Access to health care	“I talked about the delays and the ride and the authorizations, but at least they get here, right? They eventually get here.” (Radiation oncologist C)
Meeting social determinants of health	“I think there’s an unfortunate truth that being incarcerated facilitates treatment in some ways. There are patients who are lost to follow up often in the community. And that may be because of untreated mental health disease…It might be because they have unstable housing and other social determinants of health. And we’ve had patients who, when they’re incarcerated, are engaged in treatment that when released are lost to follow up and have more challenges with treatment.” (Medical director C)

Abbreviation: CT, computed tomography.

### Barriers

#### Patients’ Acceptance of Care

Participants reported that incarcerated patients decline care for several reasons: (1) fear that treatment may prolong incarceration or the belief that declining care may lead to compassionate release, (2) distrust of care quality in prison, and (3) concerns that treatment-related weakness may increase vulnerability to exploitation. For example, medical oncologist D described a patient who declined palliative treatment to extend survival by 3 years because “the only way to get compassionate release was to forgo chemotherapy. So I feel confident he would not have made that decision, were he not incarcerated.”

Additionally, patients may decline care because engaging in care is difficult. Prison transportation is uncomfortable, may result in missed events (eg, visitors) or loss of preferred bunks, such that treatment may feel punitive. Finally, care declination occurs in the backdrop of low health literacy, poor patient and clinician communication, and nontransparent scheduling that may compromise patients’ understanding of the importance of care declined.

#### Population Health and Determinants of Health

Participants noted that incarcerated individuals disproportionately come from populations with many comorbidities and risk factors for cancer (eg, substance use) and with many adverse determinants of health, such as poor access to health care. Incarcerated individuals are often more ill than nonincarcerated patients. As one medical director stated, before incarceration, patients “don’t seek medical care for their diabetes…so they’re not seeking medical care to get their colon cancer screening. And a lot of this is related, again, to social determinants of health, poverty, access to health care” (medical director E). Incarcerated patients’ challenges accessing care in the community are so pronounced that some participants reported encouraging patients to stay in prison to complete treatment: “I have patients that commit crimes to get reincarcerated to continue to access cancer care” (medical oncologist D).

#### Care Coordination

Care coordination was the most frequently cited barrier by participants, specifically in 3 contexts that introduce delays in care, potentially compromising outcomes. First, cancer workups occur sequentially, interrupted by approvals and scheduling processes, resulting in prolonged, months-long diagnostic periods. Second, after diagnosis, prison staff are responsible for coordinating care (eg, scheduling and imaging) that is ordinarily coordinated by oncology teams, as well as transportation and security. Third, incarcerated patients with cancer face numerous barriers when released from prison to the community. Oncologists reported working hard to connect patients with community care but often lacked postrelease contact information for follow-up.

#### Communication

Participants reported 3 barriers related to communication. These subthemes demonstrate how different aspects of communication intertwine to impose barriers to care.

##### Oncologist/Patient Communication

Due to escape concerns, prisons prohibit oncologists from sharing appointment dates with patients. Consequently, patients do not know if care is delayed and may decline care because they do not know why a visit is scheduled. Radiation oncologist B stated, “It’s easier to fall between the cracks because these patients can’t advocate for themselves whatsoever.” Security restrictions also prohibit family members from attending clinic visits or clinicians from calling family, such that incarcerated patients must process complex information about their prognosis and goals of care alone.

##### Communication Between Oncologists and Prison Care Teams

Oncologists provide recommendations for symptom management and care coordination in prison but rarely know whether these recommendations are enacted. Referring to medication administration in prison, medical oncologist C stated it’s “a black box…we don’t honestly know, even though we write [it] down, what medications are actually given.” Communication is further impeded by the absence of shared electronic medical records and limited communication between prisons and oncologists.

##### End-of-Life Communication

Restrictions on communication with incarcerated patients’ families can result in aggressive end-of-life care, as few patients have health care proxies identified, and clinicians lack clarity on approved processes for contacting family. Consequently, many patients die alone. Obstetric gynecologist A stated, “I think there’s a lot of ambiguity for practitioners outside of the prison as to who is the decision-maker…prisons don’t necessarily go out of their way to share what those processes are, even though they don’t want people to die without family.”

#### Symptom Management and Supportive Care

While oncologists manage symptoms for their nonincarcerated patients, participants noted that prison clinicians must manage symptoms for incarcerated patients. Although oncologists recommend medications, prison formularies may not stock these medications (eg, specific antiemetics or opioids). Moreover, it is difficult for oncologists to determine which medications patients received and thus to titrate supportive care, and patients cannot contact their oncologists between visits for help managing symptoms.

Incarcerated patients may sometimes keep basic medications in their cell (eg, ibuprofen), but others are only accessible at fixed dispensing times. Patients cannot access as-needed symptom medications outside of these times unless admitted to the prison infirmary. Medical director C stated, “We have had patients who…forgo chemotherapy because of the challenges with managing side effects in a carceral setting.”

The prison environment, including rigid medication and meal schedules, physical restrictions, and lack of privacy, may all contribute to worse symptom management. Obstetrician gynecologist A noted “those things [adjustments to diet, clothing, and housing] that I think sometimes feel like unnecessary indignities for [all incarcerated individuals] are major barriers to survival and thriving for people who are going through cancer care.”

#### Transportation

Transporting incarcerated patients is resource-intensive (patients may require 2 guards and a vehicle for appointments) and participants frequently cited transportation as a barrier to cancer screening and treatment. Cancer treatment, particularly radiation, requires adherence to strict treatment schedules that are disrupted by unreliable transportation. Yet, transportation is often limited, requiring that prisons prioritize which patients are transported to clinics. This issue is further exacerbated by shortages of security for transportation. Medical director C stated “I will say [transportation] is one of the biggest barriers to efficient health care delivery in our prison system…that is often the rate-limiting step in getting very quick access to health care.”

To facilitate transportation, patients from multiple prisons may be grouped in vans, requiring multiple stops at different prisons and clinics before each is returned to their respective prison. “For a simple clinic visit, a patient coming from a facility that’s 2 or 3 hours away, [that trip] may kind of start at 3:00 or 4:00 in the morning, and they may not get back till 10:00 or 11:00 at night” (radiation oncologist D). Transportation may be uncomfortable, especially as patients are shackled. Collectively, transportation acts as a deterrent to patients engaging in care, particularly when they are not informed in advance about its purpose.

#### Humanity in Care

Participants described several aspects of cancer care that they felt were dehumanizing to incarcerated patients. Shackling and orange jumpsuit requirements make patients uncomfortable and less likely to accept care, while possibly invoking subconscious biases among clinicians. Oncologists specifically cited shackling during radiation or the dying process as dehumanizing. Oncologists also reported that communication restrictions and limited support services deprived incarcerated patients of support from friends and family, implicitly devaluing the importance of the care decisions they faced. Moreover, patients have no privacy during life-altering clinical discussions because guards are always present, and this lack of privacy made many oncology clinicians uncomfortable: “There are guards watching me. You can’t even have a private conversation with the patient, which can also make the patient uncomfortable because the patient probably can’t also be very honest or open about what’s going on…Just a very weird, uncomfortable dynamic” (radiation oncologist F)

#### Cross-Cutting Barriers

Participants reported 3 barriers that cut across many of the previously outlined themes. Staffing shortage*s* of schedulers, PCPs, and security and transportation staff were frequently cited as barriers to timely care as prison staff bear the brunt of care coordination. Second, prisons prioritize security over health, with security concerns taking precedence over improving care quality. Medical director C stated, “the organizational mission of a prison is not the same organizational mission of a hospital or cancer center.” Third, prison clinicians and oncologists alike cited the lack of transparency of care delivered in prison as a barrier. Medical director C stated “not [all] nurses and physicians that are working in the community always kind of understand some of the barriers, the cancer treatment availability, accessibility, symptomatic availability for someone who is incarcerated. And so it just kind of creates another barrier in that already complex delivery care system.”

### Facilitators to Care

Participants predominantly discussed barriers to care, but also briefly described 2 facilitating themes, access to health care and meeting social determinants of health. Incarcerated individuals often face barriers to care in the community, such that incarceration represents an opportunity to receive care that was previously inaccessible. Medical director F reported: “in the community, [most incarcerated patients] would have a really hard time accessing services. So they come to prison, and we treat their cancer that’s been untreated for years because they couldn’t access services in the community.” Prison was described as “the last safety net” (obstetrician-gynecologist A); medical director C specifically noted: “there are ways to leverage the criminal justice system to target public health interventions in this marginalized population while you have access to them.” In addition, incarcerated individuals may have barriers to care in the community that are addressed during incarceration (eg, transportation), enabling care that would be less accessible in the community. Obstetrician-gynecologist A suggested “an incarceration episode might be a useful interrupter” of the disruptions that prevented cancer diagnosis before incarceration.

### Focus Group for Member-Checking

Twenty-two prison medical directors and clinicians participated in the focus group; demographic characteristics were not obtained. Participants affirmed themes that were presented and did not identify new themes, but noted the following issues: PCPs often have to identify care pathways anew for each cancer diagnosis; proprietary prison electronic health records make it difficult to track care and may not enable telehealth; PCPs often defer changes in symptom management until the next oncologist visit; and transportation is complicated by incarceration program requirements, mental health issues, and security levels. One unique barrier cited was that private prisons might provide less care, like screening. For facilitating themes, participants added that incarcerated individuals deprioritize health issues like screening when at home.

## Discussion

In this qualitative study, prison medical directors, clinicians, and oncologists involved in delivering cancer care to patients incarcerated in US prisons described barriers from screening and diagnosis to treatment and end-of-life care. While some of the barriers identified reflect prison systems’ prioritization of security over health and the dehumanization of incarcerated individuals, many barriers are more easily modifiable (eg, care coordination, communication, or staffing). Identification of these barriers is important because they may be mechanisms that contribute to disparities in cancer survival and signpost opportunities for improvement.

To our knowledge, this is one of the first studies to systemically evaluate barriers and facilitators to cancer care delivery in US prisons. Similar to prior research conducted in English and Welsh prisons, we identified communication, supportive care, and transportation barriers to cancer care.^7,8^ We also documented barriers across the care continuum from screening to end-of-life care that, when addressed, are tied to better outcomes. For example, better care coordination reduces time to treatment, better symptom assessment improves survival, and better communication leads to higher quality end-of-life care.^16,17,18,19^ The barriers we identified are also likely interlinked. For instance, communication barriers limit patients’ understanding of their care plans, which likely increases care declinations and contributes to worse symptom management, likely decreasing treatment tolerance and survival.

While many of the barriers to care stem from constraints from providing health care within correctional settings, they are not insurmountable. Given that incarcerated patients cannot advocate for themselves in the same way as those in the community and that most of the barriers identified occur at the institutional or policy levels of influence, responsibility for improving care is incumbent upon the institutions involved in care delivery. Participants repeatedly emphasized their commitment to providing care that meets the same standards afforded to nonincarcerated patients. A companion study^20^ to this manuscript further explores practical strategies to improve care, despite systemic challenges such as dehumanization of patients and the prioritization of security over health. These strategies include leveraging telehealth to address transportation constraints and integrating oncology involvement earlier, facilitating direct communication between prison-based and oncology clinicians, and revisiting restrictive policies on symptom management and family engagement. The facilitating themes identified in this study demonstrate that incarceration represents a public health opportunity to deliver better care for incarcerated patients.

### Limitations

This study has several limitations. Although we obtained perspectives from 32 individuals across 16 prison systems and did not identify any additional themes during a member-checking focus group with 22 clinicians, every system is unique and care is fragmented such that we may have missed unique systems or perspectives on barriers and facilitators to care. Care may also vary even within a prison system, although we interviewed state medical directors to try to understand themes throughout systems. Given that 2 of our recruitment strategies approached participants at a conference and monthly video-conference, participants in our study may be more experienced or motivated than nonparticipants, which may color their perspectives positively or negatively; yet the community of clinicians providing cancer care in prisons is very small, and these motivated participants are likely critical for any efforts to improve care. Crucially, this study did not include incarcerated patients’ perspectives; we plan to include these in future research.

## Conclusions

In this qualitative study of prison medical directors, clinicians, and oncologists involved in cancer care delivery for individuals incarcerated in US prisons, participants identified numerous barriers to quality cancer care in prisons. This study, combined with participant perspectives on how to overcome these barriers reported in the companion study,^20^ should inform efforts to improve cancer outcomes for incarcerated individuals.

## Notes

Supplement 1. eMethods. eAppendix 1. Interview Guide eAppendix 2. Focus Group Guide eTable. Barriers and Facilitators

Supplement 2. Data Sharing Statement
