# Supplementary material for: Barriers and Facilitators to Delivering Cancer Care in US Prisons
Source: JAMA Netw Open. 2025 Oct 15;8(10):e2537646. doi: 10.1001/jamanetworkopen.2025.37646 (PMC12529214; doi:10.1001/jamanetworkopen.2025.37646)
Supplement: Supplement 1. — eMethods. eAppendix 1. Interview Guide eAppendix 2. Focus Group Guide eTable. Barriers and Facilitators [file jamanetwopen-e2537646-s001.pdf]

Supplemental Online Content

Manz CR, Nava-Coulter B, Voligny E, Gundersen DA, Wright AA. Barriers and facilitators to delivering cancer care in US prisons. *JAMA Netw Open*. 2025;8(10):e2537646. doi:10.1001/jamanetworkopen.2025.37646

eMethods.
eAppendix 1. Interview Guide
eAppendix 2. Focus Group Guide
eTable. Barriers and Facilitators

This supplemental material has been provided by the authors to give readers additional information about their work.

© 2025 Manz CR et al. *JAMA Network Open.*

eMETHODS

Participant selection. Participants were recruited via three methods. First, we emailed invitations to attendees of the American College of Correctional Physicians' (ACCP) 2023 Fall Education Conference and conducted in-person interviews with interested individuals at the conference. Second, we selected several states that varied by geographic size, population, US region, and model of correctional healthcare (see next section). Through purposive sampling, we recruited participants via email from each of the seven clinical roles, identifying eligible participants through internet searches, queries to prison systems and referrals from other study participants. Third, using snowball sampling, we recruited eligible individuals via email who were identified by the research team, colleagues or study participants as likely to have unique insights into the study questions.

Selection of state prison systems: We hypothesized a priori that characteristics of state prison and correctional healthcare systems may influence the delivery of cancer care. For example:

- Geographic size: Smaller states may be able to centralize and coordinate care more easily than larger states;
- Population size: States with larger populations may have bigger prison populations with more heterogeneous cancer diagnoses, which may enable them to make more substantial investments in prison-based cancer care (e.g., on-site radiation facilities);
- Regional variation: Regions such as the Southeastern U.S. have both higher incarceration rates and worse cancer outcomes in the general population, compared with other regions, which may influence the quality of prison-based cancer care; and
- Healthcare delivery model: States that contract with for-profit companies may have different incentives for providing cancer screening and treatment than states with state-run correctional health systems.

Thus, to obtain perspectives from clinicians involved in prison systems representing these diverse characteristics, we selected five states that varied across these domains:

- Indiana: Mid-sized state and population located in the Midwest with correctional health delivered by contracted care.
- Massachusetts: Small-sized state with a mid-sized population located in the Northeast with correctional health delivered by contracted care.
- North Carolina: Mid-sized state with a large population located in the South with correctional health delivered by a mix of direct provision from the state and contracted care.
- Rhode Island: Small-sized state with a small population located in the Northeast with correctional health delivered by direct provision from the state.
- Texas: Large-sized state with a large population located in the South with correctional health delivered by direct provision from the state.

Researcher characteristics and reflexivity. Overall, none of the investigators have been incarcerated in prison or have extensive experience providing direct care to individuals incarcerated in prison. Two investigators have experience evaluating cancer care delivery and implementing interventions to improve cancer care, while two other investigators have experience conducting qualitative research. We used a team-based approach to study design and

analysis to leverage this diversity of skills. Other researcher characteristics are unlikely to influence study findings: AAW is a non-Hispanic White medical oncologist and expert in cancer care delivery and intervention research. BNC is a bi-ethnic male sociologist with a master's degree and has multiple years of qualitative interview experience, and two years working in qualitative oncology research. CM is a non-Hispanic White male who is a medical oncologist and health services researcher. EV is non-Hispanic White and Asian female who is a research project manager. DG is non-Hispanic White immigrant and is a methodologist with multiple years of experience designing mixed methods research for cancer care delivery and related research.

© 2025 Manz CR et al. *JAMA Network Open.*

eAppendix 1. Interview Guide

Participant ID: _____________
Interview Date: ____________

Introduction:

Hi, my name is [interviewer's name]. Thank you for agreeing to participate in this interview as part of a study by Dr. Christopher Manz at the Dana-Farber Cancer Institute. We appreciate the time you are taking to share your thoughts with us.

The purpose of this study is to better understand how cancer diagnosis and treatment is delivered for incarcerated patients. Our goal is to take what we learn from you and others and condense those insights to help prison systems improve cancer care for incarcerated patients. I have a few main guiding questions, but I want to emphasize that there are no "right" or "wrong" answers. Your insights on how cancer care delivery works well and how it can be improved can be equally helpful for other prisons trying to improve care. We anticipate that up to 50 individuals will participate in this study.

Participation is voluntary. Refusal to participate will involve no penalty or loss of benefits. Reponses to this interview will not be used in another study without your permission.

This interview is expected to take 30 to 60 minutes. You will be compensated with a Visa gift card of $200 for your participation. Compensation will be sent to you via email a few days after the interview.

I would like to audio record this interview. This is simply so I can focus on our conversation and do not miss any of the feedback you provide. This conversation is anonymous and confidential; meaning that nothing you say will be linked to you as an individual and no one outside the research team will have access to the recordings. We will then transcribe and process the data to facilitate analysis. We will replace your name and location with a code. The transcription will remain anonymous, meaning we will not use your name, the name of the facility or facilities where you work, or any other information that could be used to identify you.

If you do not feel comfortable answering a question, it is ok for you to tell me that you do not want to answer. You can also choose to stop this interview at any time, and you can choose to have all records of your participation deleted.

At any time, you can contact the study lead, Dr. Christopher Manz, with any questions or concerns or about any injury related to this interview by using the information on this card. For questions about your rights as a research participant, please contact a representative of the Office for Human Research Studies at Dana-Farber Cancer Institute (617) 632-3029. This can include questions about your participation in the study, concerns about the study, a research related injury, or if you feel/felt under pressure to enroll in this research study or to continue to participate in this research study.

© 2025 Manz CR et al. *JAMA Network Open.*

Do you have any questions for me?
Do you agree to participate in this study?
I have started recording. Thank you for agreeing to participate in this study.

Demographics

1. As part of the study, we will describe the characteristics of those whom we have interviewed. These characteristics will not be stored with your responses. Will you please state your age, gender, race and ethnicity?

2. What is your role in correctional health care (e.g., medical director, primary care clinician or oncology specialist) and in what prison system do you practice (e.g., Georgia state prison or Federal Bureau of Prisons).

Topic 1: Logistics of cancer care delivery

3. We are focusing on prisons (not jails) and discussing cancer care delivery, starting with screening and diagnosis and ending with end-of-life care or survivorship. Can you please describe how cancer care is delivered for individuals incarcerated in prisonin your system along this spectrum?

Guiding questions to ensure completeness:

A. *What screening is offered and where does screening typically occur (e.g., In prison? In community facilities that are not part of cancer treatment?)? What determines who gets offered screening?*
B. *Where does diagnosis typically occur (e.g., In prison? In community facilities that are not part of cancer treatment?) and how are patients linked to cancer treatment?*
C. *Where do the following treatments primarily get delivered (e.g., in prison, at an academic hospital, at a private community clinic, etc):*
    a. *Chemotherapy*
    b. *Radiation*
    c. *Cancer surgery*
    d. *Gynecologic surgery*
D. *How is cancer treatment authorized within your system (e.g., prior authorization)? How is treatment paid for within your system (e.g., fee-for-service paid by the state? Capitated payments to certain institutions)?*
E. *How do patients access as needed symptom medications?*
F. *How is palliative care specialty care and hospice delivered, both inpatient and outpatient? Are formal palliative care and hospice services available?*
G. *Upon release from prison, are there any processes to facilitate transferring care for incarcerated patients with a cancer history to community clinicians (e.g., around insurance coverage or establishing care with a community oncologist)?*

4. I am interested in hearing about your experience overseeing, coordinating and/or treating cancer for incarcerated patients. In addition to what you have already mentioned, are there

others ways in which cancer care delivery for incarcerated patients different than providing care for similar non-incarcerated patients?

Prompt on these topics: It might be helpful to think about differences around:

- *screening*
- *diagnosis*
- *the process to initiation of cancer treatment (from the suspicion of a cancer diagnosis to actually getting treatment)*
- *treatment delivery*
- *symptom management*
- *end-of-life care and*
- *survivorship (surveillance for recurrence after completion of curative-intent treatment).*
- *the logistics of how care is delivered (e.g. transportation)*
- *how care is authorized and paid for (e.g., co-pays, prior authorization)*

If they only respond with:

A. *ways that care is worse, prompt for a response on how cancer care delivery might be better for incarcerated patients than non-incarcerated patients.*
B. *ways that care is better, prompt for a response on how cancer care delivery might be worse for incarcerated patients than non-incarcerated patients.*
C. *a description of what studies or reports say about these differences, prompt for their perception of cancer care delivery based on their experience.*

Topic 2: Barriers and facilitators to high quality cancer care

5. Please tell me about some of the things that your organization does or uses to facilitate cancer care for patients in prison.

Additional prompt: For instance, some prisons use might prioritize cancer patients for transportation, or might use patient registries to track patient care.

6. In nearly all community settings, there are barriers to care (e.g., treatment delays due to transportation difficulties) that may make cancer care more challenging. The barriers to care for incarcerated patients may be similar or different as those for patients in the community. Please tell me about barriers to cancer care for incarcerated patients under your care.

Follow-up question: Barriers to care can occur at many different levels, from the individual up to health systems and policies. What barriers come to mind at:

a. *The individual level (barriers related to the patients themselves)*
b. *The interpersonal level (barriers related to the relationship between patients and other individuals involved in cancer care, including correctional staff)*
c. *The institutional level (barriers related to the structure of prison, clinics or hospitals, e.g., staffing for transportation)*
d. *The policy level (e.g., local / state / national policies)*

© 2025 Manz CR et al. *JAMA Network Open.*

Follow-up question: We have heard from others that care coordination is a particular challenge. Can you tell me whether and how that has been the case in your patient population?

Topic 3: Utility of various care delivery mechanisms for improving cancer care for incarcerated patients

7. We have discussed many barriers to care. What tools, processes or policies might help overcome these barriers?

8. Community oncology practices often use a variety of tools to try to improve cancer care delivery, and perhaps your organization uses some of these as well. I am going to list a few common tools - if your organization uses the tool, please tell me how these might be helpful, if at all. If your organization does not use the tool, please tell me how it you think it might be helpful.
   a. Cancer care navigators (e.g., staff member dedicated to making sure patients with a cancer diagnosis receive necessary tests, appointments and treatments in a timely manner)
   b. Patient dashboards / cancer registry (e.g., a list of patients with cancer +/- ways of tracking patient care or process measures)
   c. Quality improvement processes or research focused on how to improve cancer care delivery for incarcerated patients
   d. Telehealth appointments
   e. Just prior to release from prison, a formal procedure to link cancer patients to community oncologists for continued treatment or follow-up
   f. Increased access of incarcerated patients to therapeutic clinical trials
   g. Oncologists only:
      i. Tumor boards (i.e., regular multidisciplinary meetings of medical, radiation and surgical oncologists to discuss diagnostic or treatment challenges)
      ii. Next generation genetic tumor sequencing (e.g., 100+ gene tumor mutation profiling such as Foundation One) for all patients with incurable cancer

9. Finally, some systems feel they have care delivery processes that other correctional health systems may want to emulate. Please discuss any such processes in your system.

Wrap up

10. Do you have any other thoughts that you would like to share about cancer care for incarcerated patients, especially about topics that we have not discussed that you think others would want to hear about?

11. *For medical directors:* We plan to request permission to conduct similar interviews with other individuals involved in cancer care delivery for your patients. Would you be willing

to share with us the names and organizations of an individual for each of the following specialties who might be willing to participate?

a. Primary care clinician within the correctional health organization that cares for patients in your prison system
b. Medical oncologist
c. Radiation oncologist
d. Surgical oncologist
e. Gynecologic oncologist / gynecologist

Thank you for your participation. This concludes the interview. Recording has been turned off.

12. Will you please provide your email address where we can send your $200 gift card?

© 2025 Manz CR et al. *JAMA Network Open.*

eAppendix 2. Focus Group Guide

Investigator summarizes results regarding cancer care logistics, then asks:

1. Are the findings about how cancer care is delivered generally consistent with your experiences?
2. In what ways are they different?
3. Did we miss anything that you've noticed?

Investigator summarizes results regarding barriers and facilitators to care, then asks:

4. Regarding barriers:
    a. Are the findings about barriers to cancer care for incarcerated patients generally consistent with your experience?
    b. In what ways are they different?
    c. Did we miss anything that you've noticed?
5. Regarding facilitators:
    a. Are the findings about facilitators—processes that help improve cancer care delivery for incarcerated patients—consistent with your experiences?
    b. In what ways are they different?
    c. Did we miss anything that you've noticed?

Investigator summarizes results regarding strategies to improve care, then asks:

6. Of the tools and opportunities for improving cancer care that we reviewed, which are the most promising for improving care in your care settings?
7. Are there other opportunities to improve cancer care that we have not discussed that your systems use or would considering using?

© 2025 Manz CR et al. *JAMA Network Open.*

eTable. Barriers and Facilitators

Barriers
**Patients' acceptance of care**
SQ1, Medical oncologist G:<br>"Some of the challenges are with the patient population themselves. And their, I would say apprehensions about the medical care or seeing providers for different reasons. And how that might impact, one – will they get a good care is one of their apprehensions I've always noticed. Two is, will they get the standard of care is the other apprehension that I frequently notice. The third one is, would that actually delay my sentence just because I'm getting this medical care? And that is some of – I've actually seen that sometimes. It's like, yeah, my parole is coming up, I don't want to come in to get treatment because that might actually delay things"
SQ2, Medical oncologist J:<br>"They don't wanna seem weak. They don't wanna seem debilitated… once they start getting sicker, maybe they don't want to go anymore. They – or they don't want to seem weak to get beat up, so they don't wanna go, don't wanna have a cane, don't – those kinda things are important to them."
SQ3, Medical oncologist F:<br>"Or if I have a guy who is not very nice and he goes out and he's abusive with staff – the hospital doesn't want to put up with that. And they'll say they refuse to treat him.. He's not refusing care; he's just being naughty. … These guys are – some of them are already serving life in prison because they can't follow the rules. They're not gonna follow the rules when I send them to cancer center. … it's not rare. I deal with it probably once a week. And so
SQ4, Primary care physician B:<br>"I think the other piece of it is also is what goes into going out for an outside appointment. It requires that you have to be shackled, you have to – it's you're going in shackles, you're going through the back door, you having the guard with you, you're sitting in the back. And so some people will literally say, I just don't want to go through that, I don't like having to go to an appointment in that manner. And so I'd rather – I only have a couple of months left, I'd rather just get through my time [until release from prison] and then I can go to an appointment like a regular person."
**Population health and determinants of health**
SQ5, Obstetrician gynecologist A:<br>"So I think, because of the state of access to insurance, and screening and care in our state, it is absolutely the reality that people come in to prison under-screened for a lot of things."
SQ6, Medical oncologist E:<br>"[incarcerated patients diagnosed with cancer] seemed like they were having not only asymptomatic screening needs that were being unaddressed, but clearly, symptomatic presentations of illness that were going months before being fully diagnosed."

© 2025 Manz CR et al. *JAMA Network Open.*

SQ7, Medical oncologist C:<br>"So there is a significant amount of alcohol and tobacco abuse in many patients with head and neck cancer. And we do have experiences with patients who are homeless… Ironically, there may be situations where I'll say to them… I would say it's probably better for you to stay in prison until you've completed the treatment because then at least I know you're going to have transportation to your treatment."
SQ8, Medical oncologist C:<br>"I think anyone who's in our prison population or who is sentenced was once awaiting trial. And that can be a very high stress, high turnover patient population and system that makes it very tough to do screenings. And so if you're in the awaiting trial population for a year in that jail setting, cancer screening often isn't the number one priority."
SQ9, Medical oncologist D:<br>"So we historically have a lot of folks who either have received very little healthcare or they had bad experiences in healthcare for one reason or another. And a lot of their medical knowledge comes from family or friends. And maybe there were bad outcomes for a family member or a friend for whatever reason. And that just kind of adds to just a lack of trust, maybe a lack of comfort with the medical community too. I think we definitely see from a standpoint of systemic racism, you know, lack of faith in institutions. And that really does impact the way that we can reach our patients for medical care."
**Care coordination**
SQ10, Radiation oncologist B:<br>"… the biggest [barrier] is the diagnosis staging portion, how to streamline that, because if they need three tests and there's literally a month between each test, that ends up being three to four months from thinking [the patient has] cancer to actually getting started on treatment, which is a really long time. I think that's the biggest barrier I see…"
SQ11, Obstetrician oncologist A:<br>"– I think my perspective on the screening part, when we then get to where someone has concerning or has a finding on screening that suggests that they have a malignancy, I think the processes that then you maybe imagine sort of spring into action are actually, incredibly slow sort of wheels of procedure. And so when someone has a mammogram that is abnormal, that result comes back to the facility, it goes into somebody's in-basket, and that's somebody on the primary care team at the prison. That person has to read it and internalize sort of what needs to happen next. If that person needs follow-up imaging, right, needs a diagnostic mammogram, and or ultrasound or biopsy, something else, that gets scheduled outside. So that's not something that happens in the facility. And so that request basically gets entered into the system as a request for outside care. And then the scheduling sort of challenges begin, right?... But we have had, basically, a statewide staffing shortage in the prisons for years… The Medical Director and the transport folks are often sort of in a place of like, okay, so if we're going to have custody officers to transport this person to get their diagnostic mammogram, what are we not going to take somebody to? What appointment are we going to delay or cancel in order to get them there? And so often, I think they appropriately prioritize cancer patients or patients with possible cancer, and so that happens faster than then some other appointments that might be a little bit more elective. But there's still sort of a delay introduced there."
SQ12, Medical oncologist A:<br>"We are a county hospital and we don't have all the specialties. So I don't have a thoracic surgeon. I don't even have a surgical oncologist; we only have general surgeons who operate on these cancer patients. We

© 2025 Manz CR et al. *JAMA Network Open.*

don't have radiational oncology. Until recently, we didn't have a PET scanner. … so a lot of it has to do with once we don't have a service that we offer, then [the correctional health company] will have to actually to get that specific test or … will have to send that referral [for a specialist] to another hospital. …They may not have a contract with that specific hospital, and so they would have to negotiate on a case-by-base basis, and that would cause further delays."

SQ13, Obstetrician gynecologist A:
"so there's all of these points where delays happen just in screening and diagnosis. Because then once the approved request is back to the administrators, then they have to call the schedulers at the academic center, and then it's sort of a – I think the staffing shortages at the prison sort of collide with the staffing shortages at the medical center, where it's gotten harder and slower to schedule any appointments. And I think the cancer clinics and the diagnostic procedures have maintained pretty good access, but I think between finding a time when the transport folks are available at the prison to bring someone to the facility, and then when there's an appointment available, sometimes that can be a delay."

SQ14, Medical director E:
"We don't have care coordination. We have siloed care… How is a patient that's leaving prison, who has opiate use disorder, schizophrenia, and Hepatitis C, gonna see three different providers when they leave? They're not. It's not gonna happen. "

SQ15, Obstetrician gynecologist A:
"I had a patient a couple of years ago, who I did her cone biopsy, that diagnosed her cervix cancer, and she was released the next day. … Before she left, I'd gotten an address and I'd gotten two or three different phone numbers to try to coordinate her care. I managed to reach her to tell her about her diagnosis and we made an appointment [which she missed]. And so I called, and I talked to her boyfriend, and she'd left, and he didn't know where she was, and he asked if I could reach out to her on Facebook. [Or] you can try this other number. Maybe she'll know where she is. I talked to her friend who was in the hospital with COVID at the time. The friend didn't have a better number. … I was leaving my clinic phone number and the oncology clinic phone number with everybody with a pen or a cell phone. And it ended up that I couldn't connect with the patient until she was reincarcerated."

Communication

SQ16, Gynecologic oncologist B:
"When someone is incarcerated, there are limitations placed on their ability to communicate with other people that might help them make healthcare decisions. They might have restrictions on who they can call or who they can – some inmates did have email capability, but that obviously wasn't as available as it would be in the outside world. So in terms of sort of speaking to loved ones, friends, family members about healthcare things to help them – help make decisions, as a lot of people do who are not incarcerated, it was – there were just more – there are more barriers in an incarcerated setting"

SQ17, Medical director B:
"When we say shared decision-making, they're often – their experience has been that they just do as told, right? That they don't have really a say; they may have low health literacy. So those conversations that an oncologist might have with them, they don't have their family member there to bounce off or to hear something they may not have heard. So I think those conversations with the oncologists and gathering information about their – whether it's what to expect or if it's the cancer treatments, palliative versus curative, their ability to retain that because they're under so many extra stresses when they go to that

© 2025 Manz CR et al. *JAMA Network Open.*

appointment, they're handcuffed, they're with other people, they may be quite anxious, but they also may not want to be vulnerable. You got to remember this is – they have to have safety and security and they may have to put on a tough front. So they're not able to really break down and say how scared they are with their clinician or something like that. They're with other people, they may have to put on that front. And I think it just puts them at a disadvantage for communication in general with the whole cancer treatment diagnosis survivorship process without having somebody to accompany them that's really their advocate or family or friend, that is gone in this system."

SQ18, Medical director C:

"The communication between patient and provider is also far, far more limited in the prison setting and that if you're in the community setting, you might be able to shoot your cancer doctor a message. If you're concerned, you can give them a call. Even if it's Saturday at 9 p.m., there's going to be a cancer doctor or nurse on call. In the prison setting, the communication is much more limited. You'll have access to nursing staff, which does not always mimic the culture of an outpatient cancer nursing staff. You might have access to your prison physician who might be a compassionate person but will not have subject matter expertise necessarily in the cancer that you're being treated for. If you have a question, it might be days or weeks before you can actually get that message to your oncologist… there's a lot of these other barriers, including coordinating a trip team, coordinating time with the physician. It's just one extra barrier to maintaining open communication with your provider. And then finally, any time you're having a one-on-one with your oncologist, you're still shackled. You're still with correctional officers, and so there's some questions about privacy and confidentiality. "

SQ19, Obstetrician gynecologist A:

"[Referring to a patient dying in the hospital without clinicians being able to contact family] I think, really profoundly traumatizing for care providers, caregivers in the hospital, and certainly trainees who are just I think devastated in these situations because they feel like it should – that they should have been able to do something differently and don't always recognize that sometimes the system is just too broken. And that no matter how vigorously advocate or how loudly you say whatever it is that you say, that there's not a system in place to make this work well. So I think in terms of end-of-life things, I think there's a lot of ambiguity for providers outside of the prison as to who is the decision-maker, when are you allowed to talk to someone else, and I think that the prisons don't necessarily go out of their way to fair what those processes are, even though they don't want people to die without family."

SQ20, Radiation oncologist F:

"You can't even have a private conversation with the patient, which can also make the patient uncomfortable because the patient probably can't also be very honest or open about what's going on. What if that person is being abused by the guard that followed? Just very, very weird, uncomfortable dynamic, and in order to move on, you just have to quickly forget what just happened and put those emotions aside, so you can move on to the rest of your day to other patients, but it's extremely disturbing, extremely uncomfortable."

SQ21, Radiation oncologist F:

"And then the second thing – the other thing is survivorship. And survivorship, again, is also hurt by the fact that they're not aware of their follow-up dates. I don't really know the legal implications of telling people their follow-up dates, but, I guess, from a safety perspective, it's a big deal. That's why they don't tell them. But even then, if they're not going to tell them, the prison system should be held responsible because that's a huge medical-legal liability to withhold information and not act on it, especially if it has life-and-death consequences."

© 2025 Manz CR et al. *JAMA Network Open.*

Symptom management and supportive care
SQ22, Medical oncologist C:<br>“You're in an open cell with an open toilet and it's not always a comfortable spot to deal with a lot of the side effects of chemotherapy. We had one person who actually put in for medical parole and was granted medical parole in part because he was truly unable to tolerate chemotherapy while incarcerated and was getting much worse. And upon release, was tolerating chemotherapy and actually doing much better.”
SQ23, Radiation oncologist E:<br>“They have a preset list of medications that they can receive, so they have kind of a preference list, and then any medication that's not on that list requires some sort of preapproval, or you kind of really have to petition hard, and it becomes – I guess it's a formulary list. It becomes so much of a headache that generally you just try to find something that's on the list instead of pushing too hard against the powers that be to get them something unless you feel really strongly about it, but they have this formulary list, and my understanding is that they can only get their medications at certain times of the day, and so – and I think the same thing goes with meals, so I think medications are given at certain times of day, and if you leave the facility prior to that time slot, so medications are given at 9 a.m. every day, and you leave at 7 a.m. for radiation treatment, then you may not get your medication for the day… [I say to the patient] Why didn't you get this? They said, “oh, well, I left [the prison]. I'm here [in clinic] all day, so by the time I got back, they weren't giving out medications, and I couldn't get it.””
SQ24, Medical oncologist D:<br>“Otherwise, we do try to keep folks on scheduled medication to keep symptoms controlled. Depending on the patient’s security level, depending on their reliability as a patient to take the medication wisely, not traffic. And depending on what the medication is, they could keep on person. That does not apply to narcotics or sedatives, anything of that nature. And really, like promethazine, Compazine, we wouldn’t use as a keep on person. So what they would then need to do is come up to the nursing window to request their prn at the appropriate intervals. So there’s maybe some barriers to them receiving due to – let’s say there’s lockdown or custody won't allow them to leave the dorm, those things”
SQ25, Palliative care A:<br>“If your friends are living in the general population and you get moved to a medical bed, they usually can't visit you anymore. So you really can get separated from friends. ”
**Transportation**
SQ26 Palliative care A:<br>“Going out, it can be like, do I want to be shackled to a bed to get this treatment? Or do I just want to forget it? And most people choose to be shackled to the bed to get treatment if they still want the treatment. But it's not pleasant. Yeah, being transported in a van with shackles for people who are frail and weak. They're at risk of falling, risk of ulceration, and all the other things associated with shackles.”
SQ27, Primary care physician F:<br>“We’re fully dependent on Department of Corrections for transportation. So there was two or three wheelchair vans in the state, for example. So if somebody was needing a wheelchair because they physically could not get out, transfer independently, and get into the van for transport, or even the car for transport, and they had to be transported by wheelchair, then you had to reserve that wheelchair van and make sure it was available for that appointment. Because we had three in the state. Also, if there was an – a security

issue and they didn’t have officers. They couldn’t spare officers, they had to cancel a trip because of shortage of officers, then that trip would get canceled.”

SQ28, Medical director H:

“But [cancer screening is] done erratically. And why? Because it’s cost and time consuming, particularly mammogram. Even though you send them out… that’s another challenge, time and cost, to get them out. But I feel and I see that more and more that preventive services are being done, but it’s limited. “

SQ29, Radiation oncologist E:

“Sometimes [transportation problems are] system, prison-wide training or prison-related IT issues, prison-related transportation downtime, so no prisoners are being transported at all. Other times it remains a mystery, like for example, sometimes I've been told that patients weren't put on the transportation list, so let's say they're scheduled for 20 radiation treatments, and for whatever reason, so those 20 radiation treatments are May 1st through May 20th, and then for whatever reason, the patient doesn't show up on May 1st like they're supposed to, they show up on May 10th, and so that pushes their schedule back another ten days, so those extra ten days somehow don't – aren't adequately added to their schedule, and so since they're not on the transportation list, no one brings them to treatment.”

Humanity in care

SQ30, Medical director C:

“And then finally, any time you're having a one-on-one with your oncologist, you're still shackled. You're still with correctional officers, and so there's some questions about privacy and confidentiality. There's frankly some likely – just the image of being in a jumpsuit with shackles affects the provider team of implicit biases that they may or may not be aware of in treating someone who's incarcerated.”

SQ31, Radiation oncologist F:

“I saw a man who was handcuffed by hand and feet to the radiation table with guards outside the radiation door in the middle of clinic, when there are other patients there clearly uncomfortable. But even more bothersome is the patient is already on the radiation table, yet still handcuffed to the table. And so it’s a very unpleasant and inhumane treatment.”

SQ32, Obstetrician gynecologist A:

“In that, I think many of our patients come in wildly under screened, and then while they are incarcerated, may actually be over screened. And by that I just mean, the protocol has been for years that if someone comes into the system, they get a pap smear. They could have had a hysterectomy 40 years ago for benign indications, no history of dysplasia, and suddenly they have an ASC-US pap. And the chances that they have cervix cancer are actually zero because they haven't had a cervix for 40 years, but they're getting a pap smear. So that is sort of the screening piece, is that I think the system, from my perspective, is sort of so focused on not missing people and really just making sure that everyone is screened, is not always the most kind of trauma-informed approach, because it results in a lot of pelvic exams that people might otherwise not have needed. ”

SQ33, Radiation oncologist F:

“And so from the very start of symptoms, workup, diagnosis, follow up, side effect, support during treatment, it’s a complete zero. It’s almost as though we’re harming them actually by putting them through treatment, even if it’s for a cure because we’re not doing it the right way or a just way.”

Staffing

SQ34, Radiation oncologist D:

© 2025 Manz CR et al. *JAMA Network Open.*

“So it takes twice as many people to get an inmate to a clinic visit. … That's not even talking about the guards, … they have their own kind of hierarchy and structure and requirements too”

SQ35, Medical oncologist A:

“Because then once the approved request is back to the administrators, then they have to call the schedulers at the academic center, and then it's sort of a – I think the staffing shortages at the prison sort of collide with the staffing shortages at the medical center, where it's gotten harder and slower to schedule any appointments….I mean, because these systems are so arcane and esoteric and strange. ”

Prioritization

SQ36, Medical director C:

“I think in the prison setting, there's almost a penalty to implementation on other barriers to uptake [to new screening recommendations], whether it's lack of CME funding for providers to stay up to date on guidance or just getting security buy-in of like, this is a new thing we're going to have to start doing is getting people abdominal ultrasound for aortic aneurysm screening. And that's going to be a burden on the system, including correctional officers.”

SQ37, Obstetrician gynecologist A:

“But are also, I think, really profoundly traumatizing for care providers, caregivers in the hospital, and certainly trainees who are just I think devastated in these situations because they feel like it should – that they should have been able to do something differently and don't always recognize that sometimes the system is just too broken. And that no matter how vigorously advocate or how loudly you say whatever it is that you say, that there's not a system in place to make this work well. ”

Facilitators

Access to healthcare

SQ38, Gynecologist oncologist A:

“[Incarcerated patients] are the only group in the United States that's guaranteed care. That part is easier in the sense that, from a financial standpoint, it should not affect their ability to get care. We certainly know that it can affect [non-incarcerated patients]. That part is easier. I mean, they're the only ones that constitutionally are guaranteed care.

SQ39, Obstetrician gynecologist A:

“I think that the prioritization that the system does place on cancer care, and the sort of recognition of the importance of timely cancer care, I think is helpful, and reflects an understanding that it is time-sensitive care, and that it needs to happen.”

SQ40, Medical director F:

“And our benchmark is probably honestly a community standard, but I will argue that our patients get far above the community standard because of that risk of viability of we want to make sure there’s no perception that folks aren’t getting what they need. We also – because there’s payment is a nonissue, and [our state] is not a Medicaid expansion state, so we’re one of the only three states left that haven’t expanded Medicaid, so getting services for most people in my – my patient population in the community would have a really hard time accessing services. So they come to prison and we treat their cancer that’s been untreated for years and years and years because they couldn’t access services in the community. So that’s one thing that is really hard is the degree they’re coming to me sick is just absolutely horrific sometimes. I mean they’re coming in with metastatic cancer everywhere at intake and dying before they get out of my diagnostic unit because they’re – nobody treated them for up to that point. Partly because they were homeless or couldn’t access services, or unemployed, or whatever. So in some ways, like I said, I would

© 2025 Manz CR et al. *JAMA Network Open.*

argue that our patients get far better care just because payment is a nonissue in a state where payment is a huge problem.“

SQ41, Radiation oncologist F:
“And there’s an idea that – it’s actually very disturbing to think – but someone did suggest that it’s almost as though being incarcerated was a protective factor because you had better access, but that’s really disturbing. But I don’t think that’s also necessarily true. Even if you have access, the quality of care is not the same. And so the outcomes are not equal to the outcomes of you still being in the community.”

Meeting social determinants of health

SQ42, Radiation oncologist E:
“When patients don't have insurance, they can't establish care with a provider, or a provider who takes whatever sort of insurance or emergency insurance that they may have may be located several miles, several hours away from where they're living. … And then if patients have extenuating social circumstances, they may not be able to show up every day on time. And so you can take the same patient, and then they're incarcerated, then all of a sudden, they do have a way of paying for their coverage. They do have a way of getting treatment on a day-to-day basis. So you kind of remove some of these social hurdles that otherwise could have impeded care.”

SQ43, Obstetrician gynecologist A:
“I mean, honestly, as I'm saying all of this, I think what's hard is that the facilitators that exist all sort of feel like inadequate stopgap measures. So they're there, but they're often, themselves, limited or fragmented and/or otherwise complicated in terms of what is really facilitating care.”

SQ44, Radiation oncologist B:
“My patients spend a lot of time talking to me about being able to afford gas to get here. So that – I mean, I hate to say that that's easier. I'm sure the van ride is uncomfortable and that stuff. But they don't have to worry about – again, this is another patient navigation meeting. We talked about finding childcare, having a caregiver bring them, finding a ride to treatment, affording gas for treatment. So once we get the authorizations in place, the patients are usually going to show up for things. So I think that – and then for these patients again, and that's how they do fall through the cracks when they're released as well because they lose that infrastructure.”

Footnote: SQ# is the supplemental table quote number.

© 2025 Manz CR et al. *JAMA Network Open.*
