# Supplementary material for: Barriers and Facilitators to Delivering Cancer Care in US Prisons
Source: JAMA Netw Open. 2025 Oct 15;8(10):e2537646. doi: 10.1001/jamanetworkopen.2025.37646 (PMC12529214; doi:10.1001/jamanetworkopen.2025.37646)
Supplement: Supplement 2. — Data Sharing Statement [file jamanetwopen-e2537646-s002.pdf]

Data Sharing Statement

Manz. Barriers and Facilitators to Delivering Cancer Care in US Prisons. *JAMA Netw Open*. Published October 15, 2025. doi:10.1001/jamanetworkopen.2025.37646

Data
Data available: No

Additional Information
Explanation for why data not available: Individual level transcripts contain identifiable information and cannot be made available to others.
